# Changes in total homocysteine levels after acute stroke and recurrence of stroke

**DOI:** 10.1038/s41598-018-25398-5

**Published:** 2018-05-03

**Authors:** Zhihong Shi, Shuling Liu, Yalin Guan, Meilin Zhang, Hui Lu, Wei Yue, Biao Zhang, Mingzi Li, Jing Xue, Yong Ji

**Affiliations:** 1 Department of Neurology, Tianjin Huanhu Hospital, Tianjin, China; 2 Tianjin Key Laboratory of Cerebrovascular and Neurodegenerative Diseases, Tianjin Huanhu Hospital, Tianjin, China; 3 Tianjin Dementia Institute, Tianjin Huanhu Hospital, Tianjin, China; 4 Department of Nutrition and Food Science, School of Public Health, Tianjin Medical University, Tianjin, China; 5 Department of clinical laboratory, Tianjin Huanhu Hospital, Tianjin, China; 6 Peking University School of Nursing, Beijing, China

## Abstract

It is not known how total homocysteine (tHcy) levels change during the transition from acute stroke to post-stroke convalescence or whether tHcy changes occurring after the acute period are associated with recurrence of cerebro-cardiovascular events. Levels of tHcy were measured during acute ischemia and again after three months. Patients were followed for a median of 18 (range: 12–36) months. A total of 2800 patients who had at least two tHcy measurements were enrolled between February 2012 and June 2014; 2587 patients presented with ischemic stroke and 213 presented with cerebral hemorrhage. During the follow-up period, 220 (7.9%) patients experienced another ischemic event. After adjusting for additional cardiovascular risk factors, patients with the highest levels of tHcy (fourth quartile; >15.5 μmol/L) had a 1.76-fold increased risk of a recurrence (adjusted HR: 1.76, 95%CI: 1.11–3.08) as compared to patients with the lowest levels of tHcy (lowest quartile; ≤9.65 μmol/L). Additional analysis by subgroup indicated that this correlation was only significant for patients with large-artery atherosclerosis ischemia (adjusted HR: 2.00, 95%CI: 1.13–3.55). Elevated tHcy during the convalescent phase of acute stroke was independently associated with an increased risk of recurrent ischemic stroke, especially in those patients with large-vessel atherosclerosis ischemia.

## Introduction

Accumulating data suggest elevated total serum homocysteine (tHcy) is associated with an increased risk of coronary heart disease and stroke^1–5^. Studies of patients with acute ischemia, diastolic dysfunction, and hypertension as well as large community studies have also demonstrated that elevated tHcy levels increased the risk of both all-cause and cardiovascular disease specific mortality^6–10^. By design, in all of these cases control studies, stroke “cases” are identified first and the assessment of tHcy levels follows the stroke. There is concern that the disease process itself may alter blood levels of tHcy. That is, if stroke results in an increased tHcy level after the event, then the observed elevated post stroke levels among stroke “cases” may lead to misguided conclusions regarding the role of tHcy as a risk factor for the development of the stroke. It could be, as some suggest, that elevated tHcy is an acute-phase reactant and a consequence rather than a cause of the disease process^11,12^.

On the basis of these studies, it has been postulated that an elevated tHcy level prior to an ischemic event acts as a risk factor for stroke. In two previous studies, tHcy levels have been shown to increase between the acute period and after several months of convalescence after the original ischemic event^13,14^. However, these studies included only 76–82 cases; there has not yet been a large case study of changes in tHcy levels between the acute period and the convalescent period. It is still unclear when, in relation to the ischemic event, elevated levels of tHcy represent a potentially modifiable risk factor for stroke.

The relationship between tHcy levels and the recurrence of vascular events after stroke remains inconclusive^15–18^. In our previous study, we found that elevated levels of tHcy in acute stroke were related to higher mortality. However, we did not analyze the relationship between serum tHcy levels and a recurrent ischemic event. Therefore, we conducted a prospective study of a large population of patients that suffered an acute stroke to examine changes in plasma tHcy levels between the acute period and a convalescent period after a stroke, with a median follow-up period of 18 (range: 12–36) months. This study allows us to analyze whether elevated plasma tHcy levels occurring during the convalescent period after an acute ischemic event contribute to the risk of recurrence.

## Methods

### Study design

This work is based on 2800 patients who were admitted to the neurological department at Tianjin Huanhu Hospital in Tianjin, China within 48 hours after onset of cerebrovascular symptoms from February 2012 through June 2014. All patients had a magnetic resonance imaging or computed tomography (MRI or CT) scan. Individuals were diagnosed with cerebral infarction if they had a neurological deficit of presumed vascular origin that lasted 24 hours and either a normal CT brain scan or evidence of a recent infarct in the relevant area of the brain on MRI scan. The criteria for TIA was similar, but with symptoms lasting less than 24 hours and without evidence of a recent infarct in the relevant area of the brain on MRI scan. The diagnosis of intracranial hemorrhage (ICH) was based in all cases on CT showing hemorrhage. Exclusion criteria were identical to our previous study^8^, and patients died within three months of hospital discharge were also excluded from this study Controls were selected from individuals who had regular health checks from 2012 to 2014. Those patients with a history of acute stroke, coronary heart disease, renal function impairment (serum creatinine > 133 µmol/L), or B vitamin therapy within two weeks of screening were excluded.

We enrolled 2800 patients, all of whom met the inclusion criteria and provided informed consent to participate in the study. We separated patients by ischemic subtype using the criteria described in the Trial of Org 10172 in Acute Stroke Treatment (TOAST)^19^. No patients received B vitamin therapy after the initial ischemic event, but every patient did receive either a follow-up call or subsequent clinic visit every three months. The incidence of recurrent ischemic stroke was the primary outcome measured; median follow-up time was 18 months. Seven patients, who emigrated from Tianjin or refused to revisit, were lost to follow-up and excluded from the analysis of recurrent rate and risk factors.

The severity of neurological deficit was measured with the National Institutes of Health stroke scale (NIHSS) on admission. History of stroke or TIA and history of hypertension were defined as either self-reported or based on information from previous medical records. Comorbidities are described in our previous study^8^.

### Ethics Statement

The study protocol was approved by the Regional Ethics Committee (Tianjin Huanhu Hospital Ethics Committee). All patients provided informed consent prior to participation. All methods were performed in accordance with Declaration of Helsinki.

### Blood sample measurements

Blood samples were obtained from all patients within 24 hours of admission and three months after the acute event. Patients were invited to revisit their doctors for repeat sampling during the convalescent period. They attended the clinic as outpatients after an overnight fast and provided samples for tHcy analysis. Laboratory measurements used were as described previously^8^.

### Statistical methods

The Mann-Whitney U test was used for data analysis of continuous variables and chi-square statistics was used for categorical variables. Cox proportional hazards regression analyses were used to calculate hazard ratio (HR) estimates associated with tHcy levels and recurrent ischemic stroke. A p-value < 0.05 was considered statistically significant.

## Results

Plasma tHcy levels were measured at least twice in a total of 2800 patients with an acute cerebrovascular event. The baseline characteristics of all patients according to stroke subtype are described in Table 1. In 2429 patients with cerebral infarction and 158 patients with TIA, the geometric mean ± SD tHcy level on admission was 14.4 ± 10.3 μmol/L. In 213 patients with ICH, the geometric mean tHcy level on admission was 15.6 ± 9.1 μmol/L. The difference in geometric mean tHcy level between ischemic and hemorrhagic events was not statistically significant (*p* = 0.102). After three months, the tHcy level was 14.3 ± 10.0 μmol/L in patients with ischemic stroke and 14.7 ± 9.2 μmol/L in patients with ICH. The difference in geometric mean tHcy levels between ischemic and hemorrhagic events was not statistically significant (*p* = 0.603) (Table 2). The difference in geometric mean tHcy levels in patients immediately after acute stroke and during the convalescent phase was not statistically significant (*p* = 0.341).

**Table 1:** Baseline characteristics of all patients according to stroke subtype.

	Ischemic Stroke (n = 2587)	Hemorrhage Stroke (n = 213)	*p*-value
Age (years), mean (SD)	60.7 (10.5)	57.2 (10.0)	<0.001
Male, n (%)	1811 (70.0)	135 (63.4)	0.084
NIHSS in admission, mean (SD)	4.2 (3.9)	6.7 (5.2)	<0.001
Risk factors:			
Hypertension, n (%)	1666 (64.4)	172 (80.8)	<0.001
Type 2 DM, n (%)	645 (24.9)	35 (16.4)	0.005
Hyperlipidemia, n (%)	716 (27.7)	63 (29.6)	0.552
CAD	245 (9.5)	29 (13.6)	0.005
Alcohol drinker, n (%)	466 (18.0)	43 (20.2)	0.429
Smoking, n (%)	995 (38.5)	73 (34.3)	0.226
Hyperuricemia n (%)	201 (6.6))	21 (5.8)	0.382
Low physical activity, n (%)	242 (9.4)	25 (11.7)	0.255
Obesity, n (%)	216 (8.3)	28 (13.1)	0.017
Laboratory findings:*			
TG (mmol/L)	1.44	1.34	0.888
TC	4.95	5.22	<0.001
HDL-C (mmol/L)	1.06	1.19	<0.001
LDL-C (mmol/L)	2.83	2.99	0.015
Apo AI (g/L)	1.14	1.24	<0.001
ApoB (g/L)	0.98	1.02	0.132
ApoB/ApoAI ratio	0.85	0.80	0.036
Fasting glucose (mmol/L)	5.58	5.90	0.028
hsCRP (mg/L)	1.54	2.4	<0.001

Apo, apolipoprotein; CAD, coronary artery disease; DM, diabetes mellitus; HDL-C, high-density lipoprotein cholesterol; hsCRP: high-sensitivity C-reactive protein; LDL-C, low-density lipoprotein cholesterol; TG, triglycerides; TC, total cholesterol.

*Median values.

**Table 2:** Changes in tHcy levels within three months of an acute stroke, by stroke subtype and sex.

	Ischemic Stroke (n = 2587)	Hemorrhagic Stroke (n = 213)	*p-*value
Within 3 days of stroke			
Total	14.4 ± 10.3	15.6 ± 9.1	0.102
Male, n = 1946	15.7 ± 11.3	17.9 ± 10.3	0.026
Female, n = 854	11.6 ± 6.4	11.7 ± 4.4	0.868
3 months after stroke			
Total	14.3 ± 10.0	14.7 ± 9.2	0.603
Male	15.2 ± 11.3	16.2 ± 9.6	0.318
Female	12.2 ± 5.4	12.0 ± 7.6	0.821

In 232 controls, the geometric mean tHcy level was 12.1 ± 2.5 μmol/L, which was significant lower than that observed in stroke patients.

As expected, men consistently displayed higher tHcy levels than women. At baseline, the mean tHcy concentration was 15.7 ± 11.3 μmol/L for men presenting with ischemic stroke and 17.9 ± 10.3 μmol/L for men presenting with ICH versus 11.6 ± 6.4 μmol/L for women with ischemic stroke and 11.7 ± 4.4 μmol/L with women with ICH. During the three month convalescent period, mean tHcy levels were also higher in men than in women (Table 2).

The geometric mean tHcy level was not significantly different amongst ischemic stroke subtypes according to TOAST criteria, either at the acute stage or after three months of convalescence. In ischemic stroke patients with carotid or vertebral basilar artery stenosis, at three months after stroke, the geometric mean tHcy was higher than patients without stenosis (15.1 ± 12.2 μmol/L vs.13.2 ± 6.8 μmol/L, *p* = 0.030), but there was no significant difference in tHcy levels at the acute stage (14.1 ± 9.2 μmol/L vs.13.9 ± 9.8 μmol/L, *p* = 0.704). In patients age 65 or older, there was no significant difference in the geometric mean tHcy levels at the acute stage, but at three months after stroke the geometric mean tHcy was higher in patients over 65 as compared to younger patients (15.1 ± 10.2 μmol/L vs.14.0 ± 9.8μmol/L, *p* = 0.005) (Table 3).

**Table 3:** Changes in tHcy levels within three months after acute stroke according to TOAST subtypes, stenosis, and age (μmol/L).

	number	Mean tHcy within 3 days	Mean tHcy after 3months
Age			
>65 years	859	14.5 (8.8)	15.1 (10.2)
< = 65 years	1941	14.5 (10.8)	14.0 (9.8)
TIA	158	13.7 (10.6)	12.5 (6.2)
TOAST subtype			
LAA	1614	14.6 (10.6)	14.3 (9.4)
SV	625	14.1 (9.5)	14.1 (9.2)
cardioembolism	91	13.5 (6.5)	13.6 (6.5)
others	99	14.7 (9.3)	14.9 (6.4)
Stenosis			
Yes	721	14.1 (9.2)	15.1 (12.2)
No	260	13.9 (9.8)	13.2 (6.8)

### Recurrent ischemic stroke and tHcy levels three days after acute stroke

During the follow-up period, 220 (7.9%) patients experienced a recurrence of ischemic stroke. The risk of recurrent ischemic stroke was not significantly increased in patients with elevated tHcy levels within three days of the acute ischemic event (Table 4).

**Table 4:** Association of tHcy levels by quartile within three days of acute stroke with recurrent ischemic stroke (μmol/L).

**Variables**	**Quartile of plasma tHcy levels**			
	Q1 (< = 9.46)	Q2 (>9.46,< = 11.6)	Q3 (>11.6,< = 15)	Q4(>15)
Recurrent ischemic stroke, n (N)	52 (698)	52 (704)	60 (706)	56 (685)
Crude HR (95% CI)	1	1.095 (0.75–1.61)	1.28 (0.89–1.86)	1.31 (0.90–1.91)

CI, confidence interval; HR, hazard ratio; tHcy, total homocysteine.

### Recurrent ischemic stroke and tHcy levels 3 months after acute stroke

The rate of recurrence of ischemic stroke was significantly higher in patients in with the highest tHcy levels (fourth quartile) as compared to patients with the lowest levels (first quartile) (unadjusted HR: 1.61; 95% CI: 1.10–2.36; *p* = 0.011). Similarly, the rate of recurrence was significantly higher in patients with higher levels of tHcy (third quartile) than in patients with the lowest levels of tHcy (first quartile) (unadjusted HR: 1.51; 95% CI: 1.04–2.19; *p* = 0.032). This association remained significant for patients with high tHcy levels (fourth quartile) as compared to patients with low tHcy levels (first quartile), even after adjusting for age, sex, smoking status, LDL-C level, hsCRP level, and ApoB/ApoAI ratio and the presence of hypertension, type 2 diabetes mellitus, coronary artery disease, and obesity (adjusted HR: 1.76; 95% CI: 1.11–3.08; *p* = 0.031) (Table 5).

**Table 5:** Association of tHcy levels by quartile within three months of acute stroke and association with recurrent ischemic stroke (μmol/L).

Variables	Plasma tHcy levels by quartile			
	Q1 (< = 9.65)	Q2 (>9.65,< = 11.9)	Q3 (>11.9,< = 15.5)	Q4 (>15.5)
Recurrent ischemic stroke, n (N)	48 (698)	49 (703)	62 (702)	61 (690)
Crude HR (95% CI)	1	1.05 (0.71–1.57)	1.51 (1.04–2.19)^†^	1.61 (1.10–2.36)^†^
Adjusted HR (95% CI)^*^	1	0.77 (0.42–1.40)	1.52 (0.89–2.62)	1.76 (1.11–3.08)^†^
Subgroup analysis for recurrent ischemic stroke:				
Large-artery atherosclerosis, n (N)	27 (385)	32 (407)	45 (414)	37 (395)
Crude HR (95% CI)	1	1.19 (0.71–1.98)	1.75 (1.08–2.82)^†^	1.65 (1.09–2.71)^†^
Adjusted HR (95% CI)^*^	1	1.05 (0.48–2.28)	2.11 (1.04–4.28)^†^	2.62 (1.29–5.30)^†^
Small-vessel occlusion, n (N)	10 (150)	11 (132)	8 (145)	9 (137)
Crude HR (95% CI)	1	1.34 (0.56–3.22)	0.87 (0.33–2.28)	1.54 (0.62–3.80)
Adjusted HR (95% CI)^*^	1	1.57 (0.34–5.63)	0.91 (0.23–3.54)	0.39 (0.06–2.38)

CI, confidence interval; HR, hazard ratio; and tHcy, total homocysteine.

^*^Adjusted for age, sex, smoking status, low-density lipoprotein cholesterol level, high-sensitivity C-reactive protein level, Apolipoprotein B/Apolipoprotein AI ratio, and the presence of hypertension, type 2 diabetes mellitus, coronary artery disease, and obesity.

^†^*P* < 0.05 as compared with Q1.

After analyzing the data by type of ischemia, we found that patients with large-artery atherosclerosis ischemia with high levels of tHcy (third or fourth quartile) had a significantly higher risk of recurrent ischemic stroke than patients in the lowest quartile (unadjusted HR: 1.75, 95% CI: 1.08–2.82; *p* = 0.021 or unadjusted HR: 1.65, 95% CI: 1.09–2.71; *p* = 0.039). There was no significant association between patients with high and low levels of tHcy in those patients with small-vessel occlusion associated ischemic events. Multivariate analysis also did not affect this association (Table 5).

## Discussion

Only a few studies have reported serial measurements of tHcy concentrations after acute vascular events and all of these studies had small sample sizes. Ours is the first to measure tHcy levels during both the acute phase of a stroke and again three months after the event in a large sample. Our report provides evidence of a consistent pattern of the stable mean tHcy levels within three days after an acute stroke and after three months. Only in ischemic stroke patients with stenosis did the mean tHcy increase between the acute stage to convalescent phase. The rate of recurrence of an ischemic event was significantly higher in patients in the fourth tHcy quartile than in patients in the first quartile, even after adjusting for potential clinically relevant confounders; the association was especially prominent in those patients suffering from large-artery atherosclerosis.

Two previous reports^13,14^ investigated changes in tHcy from the acute to the convalescent phase of stroke (68 days to 645 days). In both, plasma tHcy levels were lower in the acute phase than in the convalescent phase, but these studies had small sample sizes. In our study, only in stroke patients with large-artery stenosis were the mean tHcy levels were lower during the acute stage than in the convalescent phase. Howard *et al*.^20^ conducted a prospective, multicenter study to examine changes in tHcy during the 2 weeks after an incident stroke. The estimated mean tHcy level at baseline was 11.3 ± 0.5 μmol/L, which increased consistently to a mean of 12.0 ± 0.05, 12.4 ± 0.5, 13.3 ± 0.5, and 13.7 ± 0.7 μmol/Lat 3, 5, 7, and 10 to 14 days after the initial event, respectively.

Landgren *et al*.^21^ reported similar increases between the acute and convalescent periods in patients with MI, in which tHcy levels of 53 patients with MI increased significantly from a mean of 13.1 ± 4.6 μmol/L 24 to 36 hours after the onset of MI to 14.8 ± 4.8 μmol/L 6 weeks after the event. Senaratne *et al*.^22^ measured tHcy within 48 to 72 hours of admission in 62 patients with acute MI and again at 6 weeks after discharge. The mean tHcy level during the acute phase was 13.6 ± 0.98 mol/L, which decreased significantly to 12.1 ± 1.01 μmol/L at 6 weeks post MI. In a small study, Egerton *et al*.^23^ reported on 10 patients with MI who completed blood draws for tHcy analysis at 1, 3, 7, and 21 days and again at 6 months. Mean levels of tHcy started at 12.9 μmol/L and increased at days 3 and 7 then decreased at days 21 and 180.

During the convalescent period in stroke patients with carotid or vertebral basilar artery stenosis, the tHcy level was higher than patients without stenosis. The multiethnic population-based Northern Manhattan Study (NOMAS)^24^ showed that Hcy levels were independently associated with carotid plaque morphology and increased plaque area; these results are similar to those from another study of Chinese adults derived from a reference population of the Kailuan Cohort Study^25^. In that same reference population, elevated tHcy levels were associated with asymptomatic carotid artery stenosis (CAS)^26^. Our study further confirmed that carotid or vertebral basilar artery stenosis in stroke patients was related to high tHcy levels during convalescent periods. Carotid plaque formation, together with CAS, was the result of atherosclerosis, which might be caused by hyper-homocysteinemia. Hyper-homocysteinemia can produce complex changes within the blood vessel wall^27^.

Our study showed that an elevated tHcy level during the convalescent phase of stroke is independently associated with an increased risk of recurrent ischemic stroke after the index cerebrovascular event. Our findings corroborate those of other investigators indicating high plasma homocysteine levels contribute to the risk of stroke recurrence^17,28,29^. Our results are also in line with those of Del Ser *et al*.^18^ who found that tHcy levels exceeding the 75th percentile three months after an ischemic stroke was a predictor of vascular events, including stroke recurrence, acute myocardial infarction, deep venous thrombosis, and peripheral arterial disease. The incidence of other vascular illness except ischemic stroke was too low to be analyzed separately in our study. By focusing solely on ischemic stroke events, our study clearly identifies elevated tHcy levels three months after acute stroke as a risk factor for recurrent stroke, especially in the large-artery atherosclerosis stroke subtype.

In our previous study, we found that elevated tHcy levels during the acute phase of stroke were associated with higher long-term mortality, but not with recurrence of stroke^8^. In the present study, we found that tHcy levels changed between the acute phase and the convalescent phase; the tHcy levels observed during the convalescent phase were related to the recurrence of ischemic stroke. These results suggest that during acute stroke, tHcy levels are influenced by a reaction to acute illness, then after three months, return to a stable level. Only persistently high serum tHcy levels affect arthrosclerosis, carotid stenosis, and recurrence of stroke.

There are several limitations of our report. Most notably, this report, like previous reports, does not assess tHcy levels level before the stroke. Therefore, we could not conclude that tHcy increased after acute stroke. In addition, our study was strengthened by an assessment of tHcy taken from acute to the convalescent period. With this additional assessment, we would have been able to assess the proportion of the change between the acute and convalescent period. These data clearly documents the need for a prospective study in which tHcy levels prior a stroke event are coupled with sequential measures after the stroke event, both in the acute and convalescent period. Furthermore, there was no systematic follow-up on tHcy concentrations during the three-month observation period. We cannot reliably document the number of patients treated with folic acid three months after the index stroke as the advice regarding folic acid was given either to the patient or to the patient’s general physician. Intake of folic acid might have led to an underestimation of the effect of hyper-homocysteinemia; however, in three months after stroke, we did not advise patients regarding folic acid, vitamin B12, and vitamin B6.

Our study shows that elevated tHcy levels during the convalescent phase after stroke were independently associated with an increased risk of recurrent ischemic stroke after the index cerebrovascular event, especially for patients of the large-artery atherosclerosis subtype. These data suggest that the clinical interpretation of tHcy after stroke, and the eligibility for clinical trials assessing treatment for elevated tHcy levels, require an adjustment for time since stroke to properly interpret the observed tHcy levels.

## References

[1] Casas JP, Bautista LE, Smeeth L, Sharma P, Hingorani AD (2005). Homocysteine and stroke: evidence on a causal link from mendelian randomisation. Lancet (London, England).

[2] Wald, D. S., Law, M. & Morris, J. K. Homocysteine and cardiovascular disease: evidence on causality from a meta-analysis. BMJ *(*Clinical *research ed)* **325**, 1202 (2002). 10.1136/bmj.325.7374.1202 PMC135491 12446535

[3] Collaboration HS (2002). Homocysteine and risk of ischemic heart disease and stroke: a meta-analysis. Jama.

[4] Bostom AG (1999). Nonfasting plasma total homocysteine levels and stroke incidence in elderly persons: the Framingham Study. Annals of internal medicine.

[5] Iso H (2004). Serum total homocysteine concentrations and risk of stroke and its subtypes in Japanese. Circulation.

[6] Sacco RL (2004). Homocysteine and the risk of ischemic stroke in a triethnic cohort: the NOrthern MAnhattan Study. Stroke.

[7] Cui R (2008). Serum total homocysteine concentrations and risk of mortality from stroke and coronary heart disease in Japanese: The JACC study. Atherosclerosis.

[8] Shi Z (2015). Elevated Total Homocysteine Levels in Acute Ischemic Stroke Are Associated With Long-Term Mortality. Stroke.

[9] Luo JL (2017). Association between plasma homocysteine concentration and the risk of all-cause death in adults with diastolic dysfunction in a community: A 13-year cohort study. Medicine.

[10] Xu B (2017). Homocysteine and all-cause mortality in hypertensive adults without pre-existing cardiovascular conditions: Effect modification by MTHFR C677T polymorphism. Medicine.

[11] Christen WG, Ajani UA, Glynn RJ, Hennekens CH (2000). Blood levels of homocysteine and increased risks of cardiovascular disease: causal or casual?. Archives of internal medicine.

[12] Brattstrom L, Wilcken DE (2000). Homocysteine and cardiovascular disease: cause or effect?. The American journal of clinical nutrition.

[13] Lindgren A (1995). Plasma homocysteine in the acute and convalescent phases after stroke. Stroke.

[14] Meiklejohn DJ, Vickers MA, Dijkhuisen R, Greaves M (2001). Plasma homocysteine concentrations in the acute and convalescent periods of atherothrombotic stroke. Stroke.

[15] Toole JF (2004). Lowering homocysteine in patients with ischemic stroke to prevent recurrent stroke, myocardial infarction, and death: the Vitamin Intervention for Stroke Prevention (VISP) randomized controlled trial. Jama.

[16] Bostom AG, Selhub J, Jacques PF, Rosenberg IH (2001). Power Shortage: clinical trials testing the “homocysteine hypothesis” against a background of folic acid-fortified cereal grain flour. Annals of internal medicine.

[17] Boysen G, Brander T, Christensen H, Gideon R, Truelsen T (2003). Homocysteine and risk of recurrent stroke. Stroke.

[18] Del Ser, T. *et al*. Hyperhomocyst(e)inemia is a risk factor of secondary vascular events in stroke patients. *Cerebrovascular diseases (Basel, Switzerland)* **12**, 91–98, doi:47687 (2001). 10.1159/000047687 11490102

[19] Adams HP (1993). Classification of subtype of acute ischemic stroke. Definitions for use in a multicenter clinical trial. TOAST. Trial of Org 10172 in Acute Stroke Treatment. Stroke.

[20] Howard VJ (2002). Changes in plasma homocyst(e)ine in the acute phase after stroke. Stroke.

[21] Landgren F (1995). Plasma homocysteine in acute myocardial infarction: homocysteine-lowering effect of folic acid. Journal of internal medicine.

[22] Senaratne MP, Griffiths J, Nagendran J (2000). Elevation of plasma homocysteine levels associated with acute myocardial infarction. Clinical and investigative medicine. Medecine clinique et experimentale.

[23] Egerton W (1996). Serial measures of plasma homocyst(e)ine after acute myocardial infarction. The American journal of cardiology.

[24] Alsulaimani S (2013). Elevated homocysteine and carotid plaque area and densitometry in the Northern Manhattan Study. Stroke.

[25] Yang X (2014). Homocysteine and carotid plaque stability: a cross-sectional study in Chinese adults. PloS one.

[26] Jia J (2016). Homocysteine and Its Relationship to Asymptomatic Carotid Stenosis in a Chinese Community Population. Scientific reports.

[27] Faraci FM, Lentz SR (2004). Hyperhomocysteinemia, oxidative stress, and cerebral vascular dysfunction. Stroke.

[28] Zhang W (2009). High plasma homocysteine levels contribute to the risk of stroke recurrence and all-cause mortality in a large prospective stroke population. Clinical science (London, England: 1979).

[29] Bos MJ, van Goor ML, Koudstaal PJ, Dippel DW (2005). Plasma homocysteine is a risk factor for recurrent vascular events in young patients with an ischaemic stroke or TIA. Journal of neurology.

